# Uncovering dynamic evolution in the plastid genome of seven *Ligusticum* species provides insights into species discrimination and phylogenetic implications

**DOI:** 10.1038/s41598-020-80225-0

**Published:** 2021-01-13

**Authors:** Can Yuan, Xiufen Sha, Miao Xiong, Wenjuan Zhong, Yu Wei, Mingqian Li, Shan Tao, Fangsheng Mou, Fang Peng, Chao Zhang

**Affiliations:** 1 Industrial Crop Research Institute, Sichuan Academy of Agricultural Sciences, Chengdu, 610300 China; 2 National Key Facility for Crop Resources and Genetic Improvement, Institute of Crop Science, Chinese Academy of Agricultural Sciences, Beijing, 100081 China; 3 Cancer Institute of Integrated Traditional Chinese and Western Medicine, Zhejiang Academy of Traditional Chinese Medicine, Tongde Hospital of Zhejiang Province, Hangzhou, 310012 Zhejiang China; 4 Comprehensive Experimental Station of Cheng Du, Chinese Materia Medica of China Agriculture Research System, Chengdu, 610300 China

**Keywords:** Evolution, Genetics, Plant sciences

## Abstract

*Ligusticum* L*.*, one of the largest members in Apiaceae, encompasses medicinally important plants, the taxonomic statuses of which have been proved to be difficult to resolve. In the current study, the complete chloroplast genomes of seven crucial plants of the best-known herbs in *Ligusticum* were presented. The seven genomes ranged from 148,275 to 148,564 bp in length with a highly conserved gene content, gene order and genomic arrangement. A shared dramatic decrease in genome size resulted from a lineage-specific inverted repeat (IR) contraction, which could potentially be a promising diagnostic character for taxonomic investigation of *Ligusticum*, was discovered, without affecting the synonymous rate. Although a higher variability was uncovered in hotspot divergence regions that were unevenly distributed across the chloroplast genome, a concatenated strategy for rapid species identification was proposed because separate fragments inadequately provided variation for fine resolution. Phylogenetic inference using plastid genome-scale data produced a concordant topology receiving a robust support value, which revealed that *L. chuanxiong* had a closer relationship with *L. jeholense* than *L. sinense*, and *L. sinense* cv. Fuxiong had a closer relationship to *L. sinense* than *L. chuanxiong*, for the first time*.* Our results not only furnish concrete evidence for clarifying *Ligusticum* taxonomy but also provide a solid foundation for further pharmaphylogenetic investigation.

## Introduction

Comprising numerous economically important species, Apiaceae has attracted increasing attention^1^. Over the past decades, extensively valuable progress has been made in evolution, phylogeny and systematics based on the investigation of morphology^2^, molecular barcode^3–5^, and whole genome sequence^1^, among others. Encompassing ca. 60 species^6^, *Ligusticum* L*.* one of the largest genera in Apiaceae, belongs to the subtribe Seselinae, tribe Ammineae, and subfamily Apioideae of Apiaceae and is widely distributed in alpine belts, meadows and forests of the Eurasian continent and North America, especially the Himalayas and North America, the two diversity centers^7,8^. However, the evolutionary scheme and the circumscription to putatively allied genera of *Ligusticum* have been a long-standing debate^3,9–14^. Based on the preceding research, a strongly supported phylogenetic implication and unambiguous classification framework of *Ligusticum*, with paramount importance in deciphering the evolutionary history of Apioideae, is predominantly hampered by the diversity of various diagnostic characters and the insufficiency of effective molecular data sets^7,9^.

*Ligusticum* comprises famous traditional oriental medicinal herbs, the bulk of which contain high amounts of natural active compounds^15^, such as alkaloids (ligustrazine)^15–18^, phenolic acids (ferulic acid)^15,17,18^, phthalide lactones (ligustilide)^19^ and volatile oils^15,17,18^, having prominent pharmaceutical values, represented by *L. chuanxiong*^17,18^, one of most pivotal medicinal plants. Owing to the clinical efficacy in the treatment of headaches, dysmenorrhea, menstrual disturbance, stroke, and cardiovascular and cerebrovascular diseases^15,17,18^, the dry rhizomes of *L. chuanxiong* (known as Chuanxiong Rhizoma or Chuan-Xiong) are extensively utilized in China, Korea and Japan. However, it is worth noting that the original plant of Chuan-Xiong was first defined by Qiu^20^ as *Ligusticum* chuanxiong Hort, a horticultural scientific name, because hitherto, exclusively extant plants were cultispecies, and they were mainly cultivated in Sichuan, China, following the extinction of wild species. The original plant of Japanese Chuan-Xiong (called “Senkyu” in Japanese) is *Cnidium officinale* Makino^21^, which was initially placed in the genus *Cnidium* but was then clustered into *Ligusticum* by Kenji Kondo et al*.*, based on the *rbcL* sequence^21^ and was further revised into *Ligusticum* and named as *L. officinale*^22^ according to universal proof based on molecular evidence^23,24^ (*L. officinale* is used hereafter). Moreover, according to the ancient records that *L. officinale* was first introduced from China to Japan in the Edo era, and based on the sequence analysis of ITS and 18S rRNA^23^, recent studies proposed that *L. officinale* was basically synonymous to *L. chuanxiong* but incongruent with *trnK*^24^. Likewise, our recent research revealed that *L. officinale* is not closer to *L. chuanxiong* than to *L. jeholens*e^25^, which also belongs to *Ligusticum*, has the trivial name LiaoGaoBen or HuoGaoBen, and is widely distributed in northern China and Korea^15,26^. In addition, *L. jeholens*e together with *L. sinense*, are the original plants of another well-known traditional medicine, GaoBen^15,27^. Although numerous studies based on morphology^28^, karyotype^29^, and mini-barcodes stated that *L. sinense* is the wild species of *L. chuanxiong*^26^, in Oriental Medicine practices, the explicitly specific property differentiation in channel tropism and therapeutic effect were indicated^30^. In herbal markets, *L. sinense* is frequently mistaken in folk medicine and is even deliberately mixed in commercial products of Chuan-Xiong due to its indistinguishable flavor and features of *L. chuanxiong* using traditional identification methods. Therefore, the issue about whether *L. chuanxiong* and *L. officinale* have a close relationship to *L. sinense* urgently deserves further exploration. Unfortunately, three additional Chinese endemic herbs are also locally known as Chuan-Xiong increasing challenges to authentication^29^. Even though their rhizome is highly similar to that of *L. chuanxiong* but with a lower quality compared to *L. chuanxiong*, according to the records of ancient Chinese medicine classics and experiences from practical application^31^. Two of those three, original plants heretofore are recognized as different cultivated accessions of *L. chuanxiong*, and one is named *L. sinense* cv. Fuxiong, which has been reported to be a triploid plant derived from *L. chuanxiong*. Nevertheless, the phylogenetic relationship among them is still unknown as incompatible frameworks were presented using data derived from karyotypes^29^, pollen morphology^28^, chemical components and DNA fragments^32^. Similarly, the subtle distinction of *L. jeholens*e and *L. tenuissimum* (Korean name, Go-Bon)^33^ resulted in improper utilization and counterfeit medicines being sold frequently in China and Korea. So far, a few complicated methods have been proposed, including multiplex PCR^24,33^ and high-performance liquid chromatography^34^ for precise identification, which depends on stringently experimental conditions. Generally, the reliable phylogeny implications and accurate and effective plant identification for those species, in this context, has become progressively imperative. Not only is it critical for promoting market supervision and improving the safety and quality of TCM (traditional Chinese medicine) but it is also of great benefit in elucidating the evolutionary event of *Ligusticum*.

Chloroplasts (CP), one of the most crucial organelles for photosynthesis in plants^35^ excluding a few algae, saprophytes and parasitical species, show a semiautonomous proliferation-deduced origin from cyanobacterium in a universally accepted endosymbiotic event^36^ and contain almost all necessary components regarding autosynthesis. In angiosperms, the chloroplast genome (CP genome) is predominately uniparental inheritance, amplified mainly through ameiosis replication with infrequent recombination^37^, and exhibits a quadripartite structure within a molecular framework ranging from 115 to 165 kb^38^. In general, the CP genome contain 110–130 unique genes of which the gene contents, gene order and genome structure are conserved^39^. Recently, the CP genome has been increasingly demonstrated able to provide sufficient variations, whereby showing high resolution in plant classification superior to that of mini-barcode fragments^40^. In past decades, phylogenomic approaches to clarify contentious phylogenetic relationships have been successfully employed: for instance, at high taxonomic levels, the phylogenetic relationship of basal lineages of angiosperms^41^ and the controversy over tree topology of the extant four orders in gymnosperms^42^ have been settled; at low taxonomic levels, the relationship of wild and domesticated rice has been resolved^43^. Moreover, utilizing the CP genome promotes the authentication in a variety of TCM that seem indistinguishable via traditional methods and have now well-discerned based on the CP genome^22,44^.

Hence, in present study, to address foregoing issues, CP genomes of the aforementioned seven species, which are valuable herbal plants in the *Ligusticum* genus, were sequenced and utilized. Our principal aims herein were to: (1) scrutinize the evolutionary dynamics of the seven plastomes within *Ligusticum* by examining the genome organization, gene content and sequence divergence to shed light on evolutionary patterns among plastomes of *Ligusitcum*; (2) identify highly variable candidate regions for species discrimination and population genetic study of *Ligusitcum*; (3) infer phylogenies to better understand the relationships of *Ligusticum* species as well as contribute to pharmaphylogenetic investigation.

## Results

### Plastome features

Seven assembled plastomes of *Ligusticum* exhibited a typical quadripartite structure with one large single-copy region (LSC) and one small single-copy region (SSC) which were separated by a pair of IRs amenable to the nature of most CP genomes in angiosperms. The CP genomes ranged in size from 148,275 bp for *L. sinense* to 148,564 bp for *L. chuanxiong* cv. Gansu and resulted from the associated length difference in four parts: LSC, from 93,682 bp for *L. sinense* to 94,012 bp for *L. chuanxiong* cv. Gansu; SSC, from 17,607 bp for *L. officinale* to 17,629 bp for *L. jeholens*e; IRs, from 18,463 bp for *L. jeholense* to 18,484 bp for *L. sinense* (Supplementary Table S2). Although a slight size difference occurred among these CP genomes, two huge deletions were specifically observed in the LSC of *L. sinense,* prominently responsible for its genome contraction (they were verified together with other Indels of a size greater than 20 bp based on PCR amplification and Sanger sequencing, Supplementary Table S3). The overall GC content of these species was almost equivalent and showed an uneven distribution across the whole CP genome with an average GC content of approximate 37.60% that was most enriched in IRs from 47.78 to 47.80% followed by LSC, about 35.99%, and lowest in SSC, from 31.09 to 31.14%, which imputed the location of transfer RNA (tRNA) and ribosomal RNA (rRNA), of which the GC content reached 55% (Supplementary Table S4). In addition, a similar GC percentage ~ 46.79% in homologous protein-coding regions (CDS) among these species was discovered to be consistent with the identity of the overall GC content. Within CDS, the AT content of each position in the triplet codon displayed the canonical bias of the CP genome, distinguished from nuclear and mitochondrial DNA^45^ that a higher AT percentage was observed at the third position in involved species, up to 70.30%, along with a sharp decrease in the second and first positions (Supplementary Table S4).

**Figure 1:**
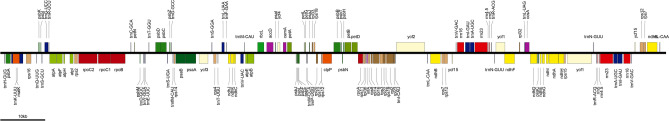
The CP genome map of seven *Ligusticum* species. Genes showed above the line are transcribed forwardly, while those beneath are transcribed reversely. Genes belonging to different functional classes were color-coded.

Compared to noncoding regions, coding sequences accounted for ~ 56.90%, showing more conserved features that encoded an identical set of 126 functional genes (Fig. 1), of which 113 were unique, harboring 79 protein-coding genes, 30 tRNA and 4 rRNA, with a coincidence of genomic organization in terms of the gene order and orientation (Table 1). Of these, 13 genes were duplicated, including three protein-coding genes, four rRNA genes and six tRNA genes, all of which resulted from IR duplication. Similar to many angiosperms, introns were discovered in 19 genes comprising 12 protein-coding genes and 6 tRNA genes. Among them, *clpP*, *ycf3* and *rps12* contained two introns, especially *rps12*, a trans-spliced gene, of which the 5′ end was located in the LSC region, whereas two replicated 3′ ends were contained within IRa and IRb regions, respectively. In addition, *trnK-UUU* possessed the longest intron that contained *matK*.

**Table 1:** List and functional classification of genes encoded by the seven genomes.

Category	Gene name
Photosystem I	*ycf3*^a^ *ycf4 psaA psaB psaJ psaC psaI*
Photosystem II	*psbC psbB psbA psbD psbH psbI psbJ psbK psbL psbM psbN psbT psbE psbF psbZ*
Cytochrome b6/f	*petB*^b^ *petD*^b^ *petG petL petN ccsA petA*
ATP synthase	*atpI atpF*^b^ *atpH atpA atpB atpE*
Rubisco	*rbcL*
NADH dehydrogenase	*ndhA*^b^ *ndhB*^b,c^ *ndhC ndhD ndhE ndhF ndhG ndhH ndhI ndhJ ndhK*
Large subunit ribosomal proteins	*rpl14 rpl16 rpl2 rpl20 rpl22 rpl23 rpl32 rpl33 rpl36*
Small subunit ribosomal proteins	*rps11 rps12*^a,c,d^ *rps14 rps15 rps16*^b^ *rps18 rps19 rps2*^b^ *rps3 rps4 rps7*^c^ *rps8*
RNAP	*rpoA rpoC2 rpoC1*^b^ *rpoB*
other protein	*accD clpP*^a^ *cemA matK infA ccsA*
Protein of unknown function	*ycf1*^e^ *ycf2 ycf15*^e^
Transfer RNA	*trnA-UGC*^b,c^ *trnC-GCA trnD-GUC trnE-UUC trnF-GAA trnfM-CAU trnG-GCC trnG-UCC*^b^ *trnH-GUG trnI-CAU*^b^ *trnI-GAU*^c^ *trnK-UUU*^b^ *trnL-CAA*^c^ *trnL-UAA*^b^ *trnL-UAG trnM-CAU trnN-GUU*^c^ *trnP-UGG trnQ-UUG trnR-ACG*^c^ *trnR-UCU trnS-GCU trnS-GGA trnS-UGA trnT-GGU trnT-UGU trnV-GAC*^*c*^* trnV-UAC*^*b*^* trnW-CCA trnY-GUA*
Ribosomal RNA	*rrn16S*^c^ *rrn23S*^c^ *rrn4.5S*^c^ *rrn5S*^c^

^a^Genes containing two introns.

^b^Genes containing a single intron.

^c^Duplicated genes in the IRs.

^d^Genes own two independent transcription units.

^e^Pseudogenes.

### Codon usage and RNA editing sites

Since usage bias of synonymous codons is widespread in organisms, it plays a vital role in evolution. Knowledge of codon preference could greatly help in understanding the selection pressure on gene expression^46,47^ and improve the translation efficiency using major codons^48^. Here, beyond the major initiator codon, in these seven species, alternative start codons were discovered in two distinct genes where ACG was used as a start codon for *ndhD* and GTG for *rps19*. Using alternative codon initiating is a ubiquitous phenomenon in eudicot plants, while previous reports also pointed out that RNA editing could restore ACG to the conventional start codon^49,50^. Overall, the 79 distinct protein-coding genes in each of the seven species were composed of 23,446–23,482 triplet codons. Of those encoded amino acids, in all presented species, the most abundant was leucine (4.14–4.17%), and the least abundant was cysteine (0.24%), which is similar to most of the reported CP genomes of angiosperm plants. The relative synonymous codon usage (RSCU) value analysis demonstrated that almost every amino acid with a synonymous codon showed a usage bias (Supplementary Table S5, Supplementary Fig. S1). Interestingly, A- or T-ended codons accounted for nearly half of the synonymous codons with commonly higher RSCU values in contrast to the other half that ended with C or G. Possibly, those reported preferences are driven by the mutational pressure in the A/T composition bias of the CP genome^51–53^.

RNA editing events have been proved universally in CP genomes since first reported^54^. Regarding the current CP genomes, a total of 56 potential RNA editing sites from 32 genes were predicted in each species (Supplementary Table S6). In all seven CP genomes, the event of S converting to L occurred with predominant frequency; by contrast, R converting to C occurred with the lowest frequency, which is in accordance with a previous investigation that the change of S to L becomes more frequent as the number of amino acids increases^55^.

### Repeat structure and simple sequence repeat analyses

Simple sequence repeats (SSRs), known as microsatellite sequences, consist of tandem short repeat units, ubiquitously distributed across the CP genome, mostly with the nature of uniparental inheritance and non-recombination^56^. Owing to the high degree of polymorphism, co-dominance and efficiency of amplification, SSRs are valuable molecular markers for mining population genetics and phylogenetic studies^57^. On average, 46 SSRs (from 42 to 49) with two motif types were identified in each species (Supplementary Table S7, Supplementary Fig. S2). SSR motifs presenting a heterogeneity frequency were predominantly rich in A/T bases. Of these SSR repeat units, 13% were detected in protein-coding regions. To capture the dynamic evolution of CP genomes within *Ligusticum* and Apioideae, the SSR characteristics of available CP genomes of representative plants in Apioideae were also investigated. Interestingly, C/G units had higher variability within *Ligusticum,* and from early-diverging lineage of Apioideae (*Daucus carota*) to Peucedaneae (*Angelica gigas*), SSR characters exhibited an increasing and prolonged tendency of SSRs, primarily in mononucleotide A/T rather than other motifs. Furthermore, the differences in SSR motif numbers among those species further demonstrated the potential of using cpSSR markers in genetic analysis among genera of Apioideae.

On average, 39 long repeats accounting for ~ 0.8% of CP genomes were detected in presented species (Supplementary Table S8, Supplementary Fig. S2), with an apparent species-specific distribution, and none were located in protein-coding regions. In contrast to SSRs, the number of four kinds of long repeats (forward, reverse, complementary and palindromic repeats) in *Ligusticum* and Apioideae displayed a significant change in certain species, ranging from 31 to 89 without a constant pattern. For instance, forward repeats were significantly enriched in *L. chuanxiong* cv. Gansu compared to its relatives, the proportion of large-sized repeats and palindromic repeats sharply increased in *L. tenuissimum*, and complementary repeats occasionally disappeared in *Ligusticum*. Moreover, repeats of *L. tenuissimum* were wholly distributed in adjacent regions of IR boundaries, which were likely to lead to its IR expansion as that in previous reports, many evidences showed the repeat sequence contributing to plastome structural variation.

### IR contraction and expansion

**Figure 2:**
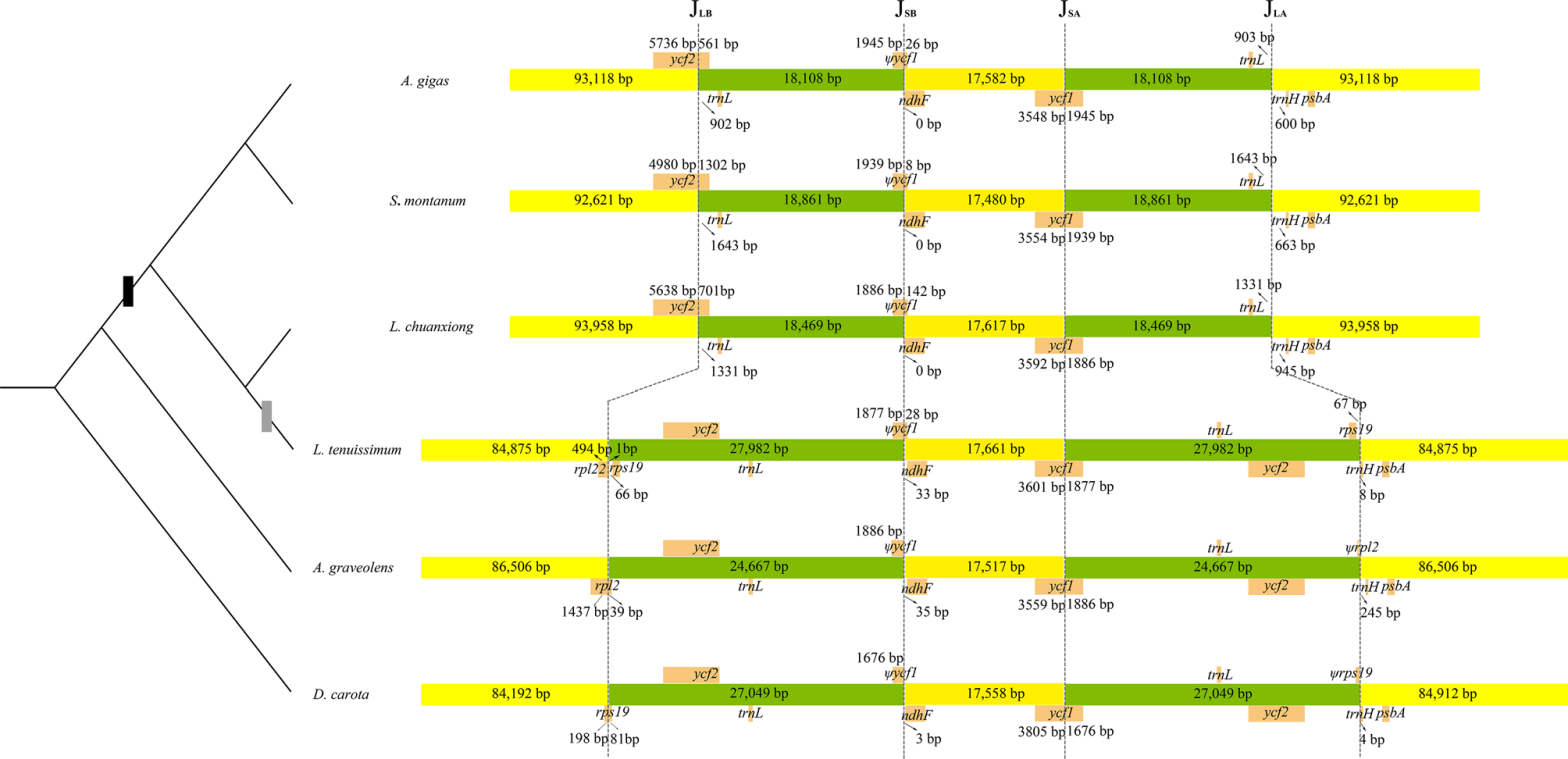
Comparison of the border position of LSC, SSC and IRs across the Apioideae. J_LB_ (IRb /LSC), J_SB_ (IRb/SSC), J_SA_ (SSC/IRa) and J_LA_ (IRa/LSC) denote the junction between each corresponding region. Genes and their locations were showed using boxes with corresponding names and with ψ representing the pseudogenes. Genes transcribed clockwise and counter clockwise are presented above and below of components, respectively. The distance between the end or start coordinate of a given gene and the border sites are indicated. The black box in phylogenetic tree denotes the branch-specific IR contraction and gray box denotes branch-specific IR expansion. These features are not to scale.

The absence of one copy of three genes, commonly duplicated and situated in the vicinity of junction sites of IR and LSC, was detected, deserving a more thorough examination. Subsequently, the evolutionary trajectories of the contraction and expansion of IR within *Ligusticum* (Supplementary Fig. S3) and Apioideae (Fig. 2) were investigated. The border of SSC/IRa crossed by *ycf1* maintained a relatively conserved state, in which a nearly constant fluctuation of ca. 100 bp was observed throughout the evolution of Apioideae. Symmetrically, the SSC/IRb boundary, located in the pseudogene fragment *ψycf1* and neighboring *ndhF,* showed an erratic shift, resulting in inconsistent deviation of the border to *ndhF* and fluctuation in *ψycf1* length. Notably, multiple dynamic expansions and contractions indicated that the junction of the LSC/IRb endpoint moved from spanning *rps19* in *D. carota* (putative ancestral IRb/LSC boundary) to *rpl2* in *Anethum graveolens*, followed by lineage-specific IR contraction to *ycf2*, causing Seselinae and Peucedaneae to lack one copy of *ycf2*, but both copies were present in *L. tenuissimum*. Considering the phylogenetic topology that *L. tenuissimum* was a sister to *L. chuanxiong,* belonging to the clade comprising species of the tribes Seselinae and Peucedaneae, and an enhanced expansion footprint in *L. tenuissimum* compared to the ancestor extending *rps19* into IR, we remain parsimonious in proposing an independent IR expansion reoccurring in *L. tenuissimum*. In addition, within the seven presented *Ligusticum* spp., an on-going shift of the LSC-IR junction and an alleviated dislocation of SSC were demonstrated whereas severe reduction hardly occurred.

Furthermore, we investigated the synonymous substitution rate of genes, *ycf2* and *rpl2* which are duplicated and de-duplicated because of IR contraction and expansion. Exceeding our expectation, the Ks value was highly variable among lineages (Supplementary Fig. S4) but without significant correlation with the copy number variation that resulted from IR contraction and expansion, which usually accelerates synonymous substitution^58,59^.

### Comparative genomic divergence and structure arrangement

**Figure 3:**
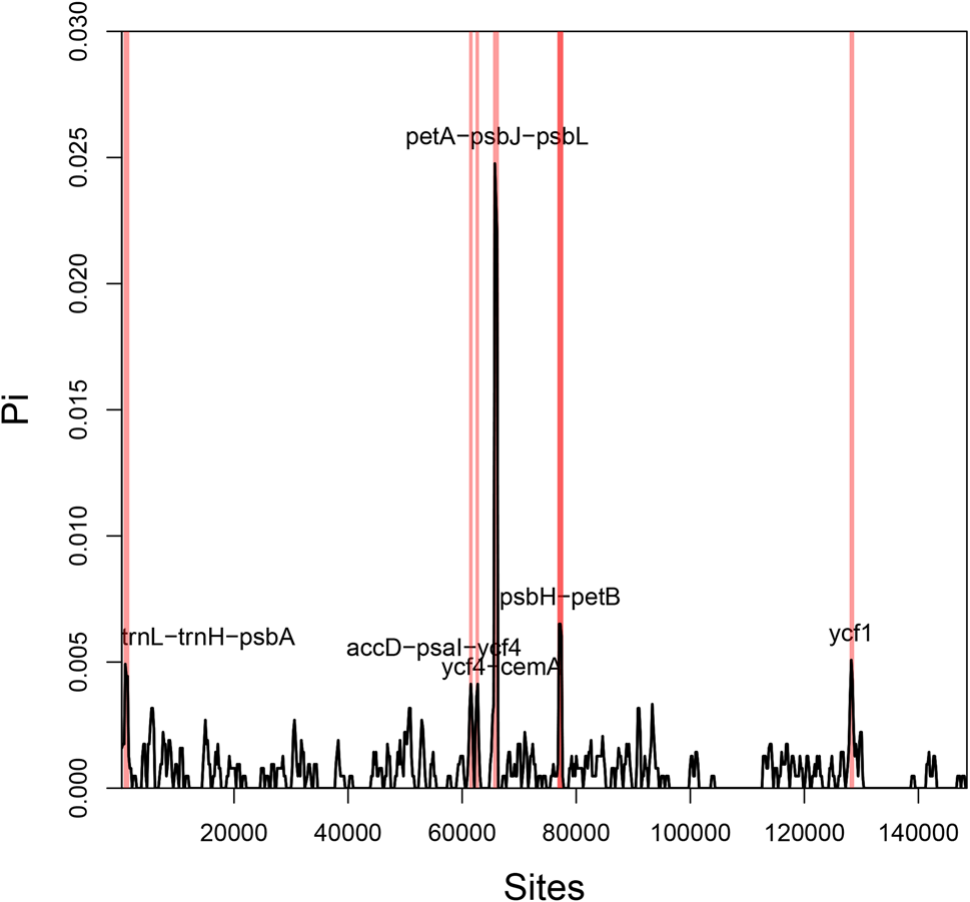
Nucleotide variability (Pi) values among plastomes. Hypervariable regions were highlighted by red shadow. X-axis: the midpoint of a window; Y-axis: the nucleotide diversity of each window.

In the view of subsequently taking full advantage of hidden mutation information in CP genomes for assisting phylogenetic inference and species identification, an in-depth investigation of the genomic structure and sequence divergence among *Ligusticum* and Apioideae was performed. Exceptionally, the CP genome sequences of *L. chuanxiong* cv. Yunnan and *L. sinense* cv. Fuxiong were identical, and the two were definitely two different species: one diploid with flowers and seeds and one triploid reproduced solely by means of vegetative propagation^29^. This result was double checked via subsequent conventional Sanger amplicon sequencing of variation hotspot regions. Synteny and sequence divergence analyses demonstrated that the seven *Ligusticum* species exhibited a high degree of colinearity (Supplementary Fig. S5) and sequence identity at the genome-scale level. The nucleotide diversity value (Pi) across the genomes ranged from 0 to 2.5% and nearly 45.6% of the compared regions showed 100% identity (Fig. 3). A higher divergence in the LSC region and lower divergence in IR were demonstrated, implying general conservatism of IR in contrast to other regions, which is in congruence with characteristics for the majority of angiosperms. Furthermore, the most divergent loci were located in *petA*-*psbJ*-*psbL* with mean Pi = 0.023, and six additionally different hypervariable regions (Pi > 0.004) were determined, including *pebH-petB*, *trnL-trnH-psbA*, *accD-psaI-ycf4*, *ycf4-cemA*, *psbH-petB* and *ycf1*, providing potential candidate regions for genus-specific barcode marker mining. Recently, increasing researches revealed wide prospects of using Indel marker derived from CP genomes for species identification and authentication of herbs^22,25,60^. Here, the insertion and deletion were detected to be located mainly in noncoding regions especially marked in *psbB-petB* and *trnM-psbD*. Intriguingly, Indels have also been found frequently occurring in *ndhB* and *ycf2* coding regions next to the boundary of IRb/LSC (Supplementary Fig. S6), which indicates intensive changes around corresponding junctions; this is compatible with common phenomena found in closely related species^61^. Despite conserved synteny in gene order and orientation of CP genomes among Apioideae (Supplementary Fig. S7), a higher sequence divergence and length variation, specifically in *trnL-psbA* and the regions between *rpoB* and *psbD*, where protein-coding genes are rarely situated, were disclosed. In contrast, the IRb/LSC boundary of Apioideae was more conserved (Supplementary Fig. S8) compared to the *Ligusticum* genus in consideration of the comparison among Apioideae using genetically distant related species. If the same underlying mechanisms within Apioideae were involved, accordingly, a high divergence would be detected.

### Phylogenetic analyses

**Figure 4:**
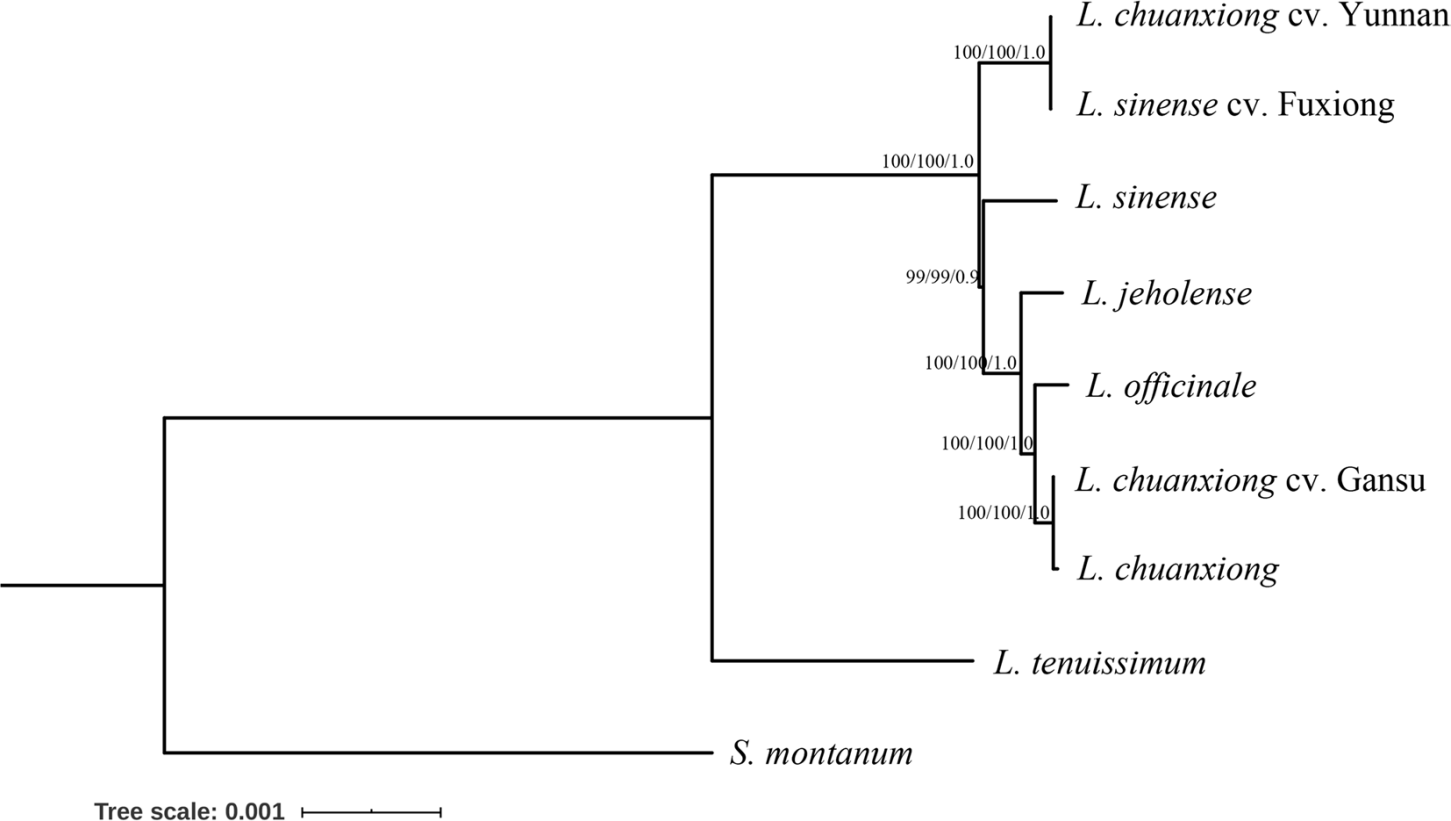
ML tree of *Ligusticum* taxa based on entire CP genomes. *S. montanum* was used as an outgroup. The numbers above each node from left to right are supported values from 1000 bootstrap replicates that were generated based on MP, and ML, and the posterior probability based on BI.

To address the relationship among the seven medicinal species, phylogenetic analysis was carried out using entire CP genome sequences because of an extreme synteny among those CP genome and limited parsimony informative characters in the CDS of 79 shared protein-coding genes where scarcely 129 sites were found. Three inference methods, including Bayesian inference (BI), maximum likelihood (ML) and maximum parsimony (MP), were employed along with *Seseli montanum* as an outgroup. The topologies generated by whole genomes were highly concordant, and almost every node was highly supported, regardless of the different methods used. Among all constructed trees (Fig. 4), *Ligusticum* comprised two separate subclades where *L. tenuissimum* presented as a sister clade to the remaining seven taxa. *L. chuanxiong* cv. Gansu and *L. chuaxiong* demonstrated a closer relationship, together forming a clade sister to *L. officinale,* receiving a robust supporting value (~ 100%) for all methods, and this entire group was clustered as the sister clade to *L. jeholense* following the clade of *L. sinense* cv. Fuxiong and *L. chuanxiong* cv. Yunnan. To further verify the phylogenetic relationship obtained, the phylogenetic signal across the genome was measured. As expected, this highly consistent tree was supported by phylogenetic signal analysis based on the value of delta site-wise log-likelihood scores (ΔSLS) with all four strong sites (absolute ΔSLS > 0.5) and 81.3% weak sites (absolute ΔSLS ≤ 0.5) favouring, however, here a lower highest value reaching at most 2.3 was observed (Supplementary Fig. S9). Additionally, extremely short branch lengths within those seven species were observed.

**Figure 5:**
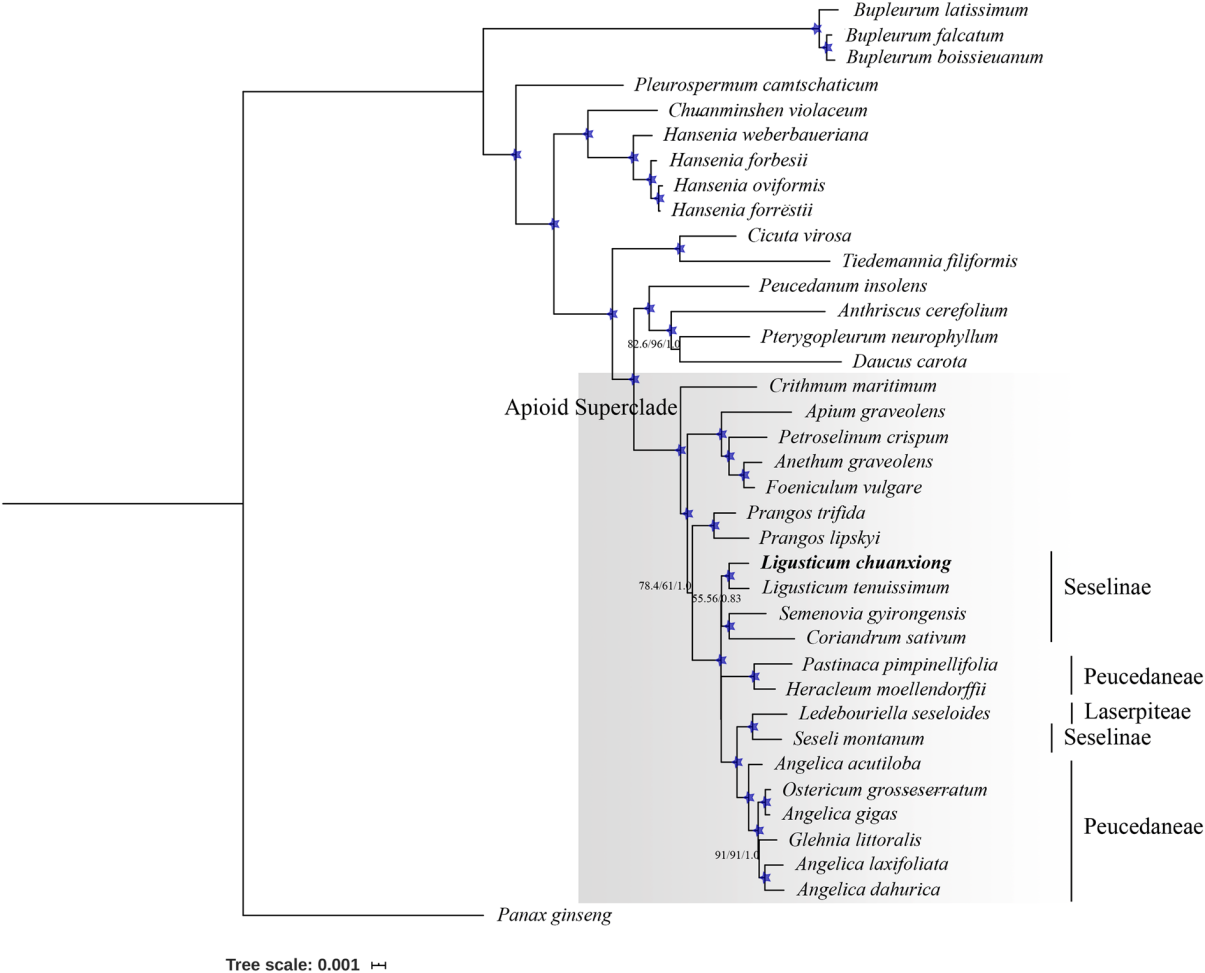
Phylogenetic topology of 36 species within Apiaceae constructed based on the CDS of 70 protein-coding gene sequences using MP, ML, and BI methods with *P. ginseng* as an outgroup. The supported value of MP, ML, and BI were shown along with each node, and stars represent a bootstrap value ≥ 99% and posterior probability = 0.99.

As yet, the increasing availability of CP genomes for Apiaceae provides us unprecedented resources to precisely clarify phylogenetic relationships and investigate the taxonomic status of *Ligusticum* within Apioideae via phylogenomic analyses. Here, based on 70 shared protein-coding sequences involving 37 species that contain 4196 parsimony informative characters and *L. chuanxiong* were used to represent those seven species that were attributed to limited parsimony information in CDS. Phylogenetic trees were constructed using maximum parsimony, maximum likelihood and Bayesian inference, with *Panax ginseng* as an outgroup. The phylogeny produced from each analysis was topologically identical, and most nodes agreed well with previous relevant plastid genome analyses within Apioideae^22,62^ (Fig. 5). Overall, 29 of 33 nodes received a maximally supported value of 100% bootstrap (BS) and 1.0 posterior probability (PP). Notably, *Ligusticum* formed a monophyletic clade that was placed within Seselinae and allied with a group comprised of *S. montanum* and *Coriandrum sativum*, with weak support in ML and MP but moderate support in BI. An ambiguous circumscription between Seselinae and Peucedaneae was depicted in the present cladogram, which is similar to earlier studies. In accordance with previous phylogeny results based on CP genomes, *Glehnia littoralis*, positioned within the genus *Angelica*, was moderately supported^22^. Moreover, two *Prangos* plants were weakly clustered as a sister clade to the group consisting of Seselinae and Peucedaneae, which requires further reexamination.

## Discussion

Since the first CP genome of Apiaceae was reported^63^, along with the rapid development of sequencing technology in past decades, approximately 50 CP genomes were figured out within Apiaceae. However, plastid sequences for *Ligusticum*, the taxonomic scheme and the placement of which is one of the most difficult genera to clarify in Apiaceae, are scarcely available. The seven newly established CP genomic sequences significantly enrich the molecular resources for *Ligusticum*. The conserved features of gene content and organization, gene orientation, and intron number among those CP genomes were revealed to be similar to the variability within previously reported species of *Camellia*^64^, *Panax*^65^ and *Epimedium*^66^. Despite the fact that a long time has passed since its divergence from *D. carota*, those genomes share an identical gene set, with a similar organization further indicating the structural conservation in contrast to Circaeasteraceae^67^. Although a higher nucleotide variability value was presented in certain divergence hotspot regions based on the diversity investigation and complete genome pairwise alignment, the entire variation exhibited a conserved tendency. Strikingly, in our study, the plastid genome sequences of two species were completely identical, which has barely been reported and may be caused by recent divergence. Overall, a moderate divergence among sequences was demonstrated, compared to several genera recently reported^44,61,68–70^. Of note, *petA-psbJ-psbL*, the most divergent region revealed in the present study, has also been demonstrated to be highly divergent in many genera^68,69,71,72^. Even though high diversity was observed in *trnH-psbA* and *ycf1*, which have extensively suggested to be taken as universal barcodes^73^ and depicted has a capacity for sufficient variation information for taxon discrimination in angiosperms^72,74^, no one hotspot alone was enough to distinguish these seven herbs. Thus, with plastid scale-level analyses, we proposed a combination strategy of those hotspot regions to enable us to definitively distinguish these species and elucidate a comprehensive resolution, which prior studies were not able to achieve based on a single fragment.

Intriguingly, in the present study, we noticed the dramatic branch-specific CP size reduction in the sub-clade consisting of Peucedaneae and Seselinae, including a considerable number of famous oriental medicinal plants. Subsequently, the lack of one copy of the gene clusters located at the boundary of IRa/LSC attributed to IR contraction was observed. Compared with the IR type of *D. carota*, *rpl2*, *rpl23*, *trnL-CAU* and *ycf2* were lost in *A. gigas*, *L. chuanxiong* and *S. montanum,* implying a branch-specific IR shift. For a certain species within this branch, multiple rounds of contraction and expansion occurred resulting in CP genome re-expansion and of which the IR is even larger than that of the ancestor type; for instance, the IR border of *L. tenuissimum* re-extended to *rpl22,* which led to a duplication of *rps19* that primitively spanned the junction in *D. carota*, and could potentially be a crucial character in *Ligusticum* taxonomy. As early as the last century, the frequency fluctuation and large size shift of IR within Apioideae, especially, within the apioid superclade, were noticed and used to reconstruct the phylogenetic relationship^75^, which re-placed *L. officinale* in the Angelica group based on the restriction map. Likewise, IR variations in Berberidaceae^76,77^, conifers^78,79^, legumes^59^, and ferns^80^ were found and used to reconstruct their phylogeny. The rapid development of sequencing technology has allowed us to establish the CP genome, which enables us to further precisely confirm and define the endpoint of IR. Indeed, the examination of IR shifts within *Ligusitcum* at nucleotide level were reported for the first time and to the best of our knowledge, such a large-scale shift within one genus has not been reported in apioid superclade. Large-scale expansion and contraction (over 1 kb) of IR across Apioideae were revealed to be primarily confined at the boundary of LSC/IRb displaying a lineage-specific flux whereas the situation of SSC/IR exhibited a constant character similar to most non-monocot angiosperms^81,82^. Recently, as the availability of plastid genomes increases, IR border shifts become more frequently reported, yet large-scale expansion and contraction of IR are considered uncommon phenomena^81^, principally observed in heterotrophs^83^ and a few autotrophs^84^. However, unlike the case of rearrangement that frequently occurred in the CP genome of which IR remarkably shifted, for instance, *Pelargonium*^85^, conifers^79,86^, *Clemati*^87^, legumes^59^, *Asarum*^88^, etc., the genome structure and gene order within the apioid superclade are significantly conserved^81^. Hence, the large-scale alteration of IR within this clade has great benefit for the study of the underlying mechanism causing large-scale expansion and contraction of IR^81^. In comparison to small alterations of IR which are supposed to result from gene conversion^89^, large-scale IR alterations are attributed to the double-strand break along with illegitimate recombination accounted by repetitive sequence possibly^75,89,90^. But herein, at the boundary of LSC/IRb of seven new plastomes, without a directly supporting evidence of those hypotheses, the repetitive motif, was observed in accordance with some previously reported plastomes in Apiaceae^81^. Nevertheless, around the junction of LSC/IRb in *L. tenuissimum*, ploy(A) and ploy(T) tracts were discovered. Furthermore, according to previous reports, a novel fragment, the derivation of which remains unsettled, was detected concomitantly residing between LSC/IRa in the CP genome of the apioid superclade and might be contributing to IR shift in Apiaceae^81^ while a ~ 500 bp insertion was recovered in our seven plastomes but was absent in *L. tenuissimum*. In *Petroselinum*, the corresponding homologous regions are highly similar to the intergenic sequence of *cob*-*atp4* of the mitochondrial genome^81^ and were postulated to be transferred from the mitochondrial genome; however, the insertion fragment of seven *Ligusticum* plant plastomes did not have a specific homologous region in mitochondrial DNA. To be prudent here, those aforementioned mechanisms that were presumed responsible for the IR shift should not be precluded without further convincing evidence. Previously, a decelerated synonymous rate of duplicated genes led by IR fluxes was uncovered^58,59^ while herein, no significant changes of the synonymous rate in *Ligusticum* was manifested, similar to the *ycf2* retention event in gingko^91^.

Combining 37 available CP genomes, a phylogenetic analysis of Apiaceae was carried out, of which the phylogenetic trees were highly similar to the topological structure that was recently reported based on CP genomes except for the node with a weakly supported value. Considering a limited variation in protein-coding regions within *Ligusticum*, phylogenetic inference of eight *Ligusticum* species was performed based on the entire genome. *L. tenuissimum* was a sister to the clade comprising the remainder, with a highly supported value, which is in line with the relationship deduced from morphological cladistic analysis using 40 characters^9^. Here, *L. chuanxiong* cv. Gansu and *L. chuanxiong* were clustered as a sister clade to *L. officinale,* verifying a closer relationship of *L. chuanxiong* cv. Gansu and *L. chuanxiong,* coincident with the origin investigation by herbal textual research, i.e., they belong to the western type of Chuan-Xiong^30^. In addition, we further confirmed the former revision and repositioning of *L. officinale* in the *Ligusticum* genus based on molecular cytogenetic^92^ and barcoding^21,23^ analyses, providing the molecular evidence for the ancient record that *L. officinale* was introduced from China. Above all, a closer relationship of *L. chuanxiong* to *L. jeholense* rather than *L. sinense* was revealed for the first time. Therefore, here, we approved the original nomenclature of *L. chuanxiong* presented by Qiu^20^ instead of the revision by Fu^26^. Previous studies based on karyotype suggested *L. sinense* cv. Fuxiong was a triploid of *L. Chuanxiong*^29^, while, as another foremost discovery, we provided the distinct result that *L. sinense* cv. Fuxiong had a closer relationship with *L. chuanxiong* cv. Yunnan and with affinity to *L. sinense* rather than to *L. chuanxiong*. We purely presume that the sequence identity of *L. sinense* cv. Fuxiong and *L. chuanxiong* cv. Yunnan resulted from incomplete lineage sorting or a recent divergence event, owing to the triploid event of *L. sinense* cv. Fuxiong, of which the ancestor probably derived from Yunnan that was demonstrated as the most diverse center of *Ligusticum* in China^7^. Furthermore, *L. sinense* cv. Fuxiong or the ancestor was introduced and domesticated in Jiangxi. Our present analyses simultaneously encompassed different original plants with nomenclatural types of *L. chuanxiong,* which are actually distinct, whereas previous researchers did not realize this. In light of the present findings, we strongly support the hypotheses that ancient Chuan-Xiong was independently derived from two regional groups of original plants with different distributions and cultivation centers, one in the north of China, including *L. chuanxiong* and *L. chuanxiong* cv. Gansu, and the other mainly in the south, including *L. sinense* cv. Fuxiong and *L. chuanxiong* Yunnan. Our data also elucidate a relatively distant relationship between *L. chuanxiong* cv. Yunnan and *L. chuanxiong* suggesting the scientific name should be revised for *L. chuanxiong* cv. Yunnan. Furthermore, we obtained phylogenetic trees based on CP inheritance and our assumption could be further scrutinized by integrating nuclear and mitochondrial data.

## Materials and methods

### Plant materials, DNA extraction and sequencing

The seven *Liguisticum* species used in this study were collected from different places. The detailed collection and identification information of each sample is listed in Supplementary Table S1. The voucher specimens were deposited in the herbarium of the traditional Chinese medicine planting center of Sichuan, Industrial Crop Research Institute, Sichuan Academy of Agricultural Sciences, China, and living plants were permanently planted in germplasm nurseries of traditional Chinese medicinal plants, at the scientific research base of the Industrial Crop Research Institute, Sichuan Academy of Agricultural Sciences, China. Fresh leaves of each plant were collected and frozen in liquid nitrogen and then surrounded by dry ice. Leaves were divided into two parts: one was used for sequencing, and the other underwent long-term storage at − 80 °C for later use. Total genomic DNA was extracted using the modified CTAB method^93^ and then the concentration and purity were examined using spectrophotometric methods by a Nanodrop-2000 spectrometer (Nanodrop Technologies, Wilmington, DE, USA) and DNA agarose gel electrophoresis by comparison with marker and reference DNA samples. A 350 bp sequencing library was prepared strictly according to the manufacturer’s instruction using highly pure DNA samples and was subsequently subjected to the HiSeq X Ten platform. At least 10 Gb data of 150 bp pair-end reads for each sample were obtained.

### Genome assembly, annotation, and sequence features

Using publicly available CP genome sources of relatives as a reference, CP genomes of seven species were assembled using NOVOPlasty 3.0^94^ with default kmer value, and the junction regions were confirmed by Sanger sequencing. The assemblies were annotated via the online website OGDRAW 1.3.1 (https://chlorobox.mpimp-golm.mpg.de/OGDraw.html)^95^ with default parameters along with tRNA identification. The annotations were manually examined and revised by comparison with homologous genes and reference genomes using several types of software: Geneious v10.2.2, MEGA 7.0^96^ and Apollo v1.11.8^97^. The linear genome map was drawn using OGDRAW. The assembled and annotated results were submitted to NCBI with corresponding accession numbers (Supplementary Table S1). The following sequence features were analyzed: (1) GC content was calculated using an in-house Perl script; (2) SSRs were detected using MISA^98^ with the setting file as follows: mononucleotide-ten, dinucleotide-six, trinucleotide-five, tetranucleotide-five, pentanucleotide-five, and hexanucleotide-five, interruptions-one hundred; (3) Codon usage bias was calculated by MEGA 7.0, and the RSCU (Relative synonymous codon usage) ratio with a threshold value of 1 was applied to estimate the usage preference of synonymous codons; (4) Direct (forward), reverse, complement and inverted (palindromic) dispersed repeats were examined via the online program REPuter^99^ with parameters as follows: hamming distance was set to 3, minimum and maximum sizes of repeats were 30 bp and 500 bp, respectively, and redundant repeats were manually removed; (5) RNA editing sites were predicted through the online program Predictive RNA Editor for Plants (PREP-Cp)^100^ with a cutoff value of 0.8.

### Genome comparison

Prank^101^ and MAFFT version 7^102^ were used for multiple sequence alignment with default parameters. The sequence identity of CP genomes was intercompared and visualized using mVISTA^103^. The annotation of *L. chuanxiong* was taken as a reference. Colinearity and rearrangement of the CP genome were determined by Mauve^104^ with default parameters. When running Mauve, *L. chuanxiong* cv Ganshu and *A. graveolens* were taken as reference for detecting within *Ligusticm* and Apiaceae, respectively. The nucleotide diversity value was calculated by DnaSP v6^105^ using a sliding window length of 600 bp and a 200 bp step size. Pairwise synonymous substitution values were examined based on bioperl and were normalized following Chaw’s method^91^. The Wilcoxon test was implemented to determine the significance level.

### Phylogenetic analysis

In total, 43 CP genomes were used for phylogenetic relationship inference, of which 36 were downloaded from NCBI (Supplementary Table S9). Two data sets were used: (1) for phylogenetic analysis among *Ligusticum*, the entire CP genome sequence was used*,* and (2) for the intergeneric phylogenetic analysis within Apiaceae, the CDS of common protein-coding gene were used. The whole genome was aligned using MAFFT version 7. The CDS of seventy common protein-coding genes were detected and extracted using an in-house Perl script, and multiple sequence alignments of each gene were executed separately via two programs, Clustal W 2.1^106^ and MAFFT version 7. Afterwards, the aligned CDS sequences were concatenated into one data set for further analysis. Phylogenetic analysis was performed based on three different algorithms, MP, ML and BI. An optimal nucleotide substitution model was implemented via the analysis of jModelTest 2^107^ and the model with the best corrected Akaike Information Criterion (AICc) value was selected. RAxML version 8^108^ was used for ML tree construction with 1000 bootstrapping replicates and the nucleotide substitution model GTR+G was token based on the results of jModelTest 2. Using PAUP version 4^109^ with a heuristic search method (repeat 1000), the MP tree was constructed and tested by the bootstrap method as well. Mrbayes v3.2.6^110^ was used for BI tree construction with at least, 2,000,000 iterations of the Markov Chain Monte Carlo method. When p values converged, the majority-rule consensus tree was constructed based on the remaining 75% of the sample. The unconstrained ML tree stated above was set as T1 for phylogenetic signal exploration while the alternative tree (T2) was obtained based on the ML constrained method referring to the framework described by Shen^111^, the method of which site-wise log-likelihood and ΔSLS was calculated. The threshold for strong and weak sites was set based on Shen’s method^111^.

## Supplementary Information

Supplementary Figures.

Supplementary Table S1.

Supplementary Table S2.

Supplementary Table S3.

Supplementary Table S4.

Supplementary Table S5.

Supplementary Table S6.

Supplementary Table S7.

Supplementary Table S8.

Supplementary Table S9.

## Data Availability

All the scripts and commands used can be found at https://github.com/can11sichuan/test/tree/master.
