# Supplementary material for: Uncovering dynamic evolution in the plastid genome of seven Ligusticum species provides insights into species discrimination and phylogenetic implications
Source: Sci Rep. 2021 Jan 13;11:988. doi: 10.1038/s41598-020-80225-0 (PMC7806627; doi:10.1038/s41598-020-80225-0)
Supplement: Supplementary file 1 — Supplementary Figures. [file 41598_2020_80225_MOESM1_ESM.docx]

# Uncovering Dynamic Evolution in the Plastid Genome of Seven *Ligusticum* Species Provides Insights into Species Discrimination and Phylogenetic Implications

Can Yuan ^1,4^, Xiufen Sha ^1,4^, Miao Xiong ^1,4^, Wenjuan Zhong ^1,4^, Yu Wei ^2^, Mingqian Li ^3^, Shan Tao ^1,4^, Fangsheng Mou ^1^, Fang Peng ^1,4,^* & Chao Zhang ^1,4,^*

^1^ Industrial Crop Research Institute, Sichuan Academy of Agricultural Sciences, Chengdu, 610300 China; [scnkyjzsxy@163.com](mailto:scnkyjzsxy@163.com) (C.Y.); shaxiufen6@163.com (X.-F.S.); xiongmiaocd123@163.com (M.X.); [wenjuanzhongsaas@sina.com](mailto:wenjuanzhongsaas@sina.com) (W.-J.Z.); [jzstaoshan@163.com](mailto:jzstaoshan@163.com) (S.T.); fshmu@163.com (F.-S.M.); prefer1134@163.com (F.P.); jychaozhang@163.com (C.Z.)

2 National Key Facility for Crop Resources and Genetic Improvement, Institute of Crop Science, Chinese Academy of Agricultural Sciences, Beijing 100081, China; [weiyu@nwsuaf.edu.cn](mailto:weiyu@nwsuaf.edu.cn) (Y.W.)

3 Cancer Institute of Integrated traditional Chinese and Western Medicine, Zhejiang Academy of Traditional Chinese Medicine, Tongde hospital of Zhejiang Province, Hangzhou, Zhejiang, 310012, China; [limingqian613@163.com](mailto:limingqian613@163.com) (M.-Q.L.)

4 Comprehensive Experimental Station of Cheng Du, Chinese Materia Medica of China Agriculture Research System, Chengdu 610300, China

* Correspondence: prefer1134@163.com (F.P.), jychaozhang@163.com (C.Z.); Tel.: +86 028 68907211 (F.P.); Tel.: +86 028 68907207 (C.Z.)

## Supplementary Materials:


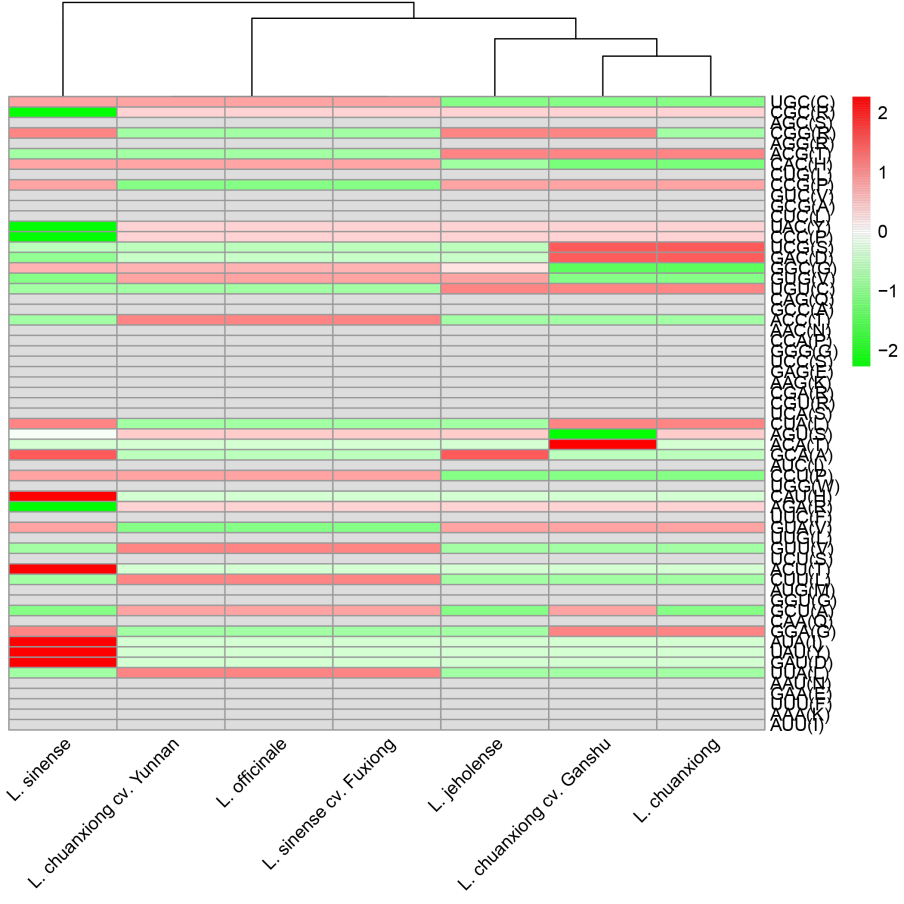


**Figure S1.** Heatmap of RSCU (relative synonymous codon usage) values of the amino acids. The variation from red to green indicates the RSCU values vary from higher to lower levels. Clustering analysis was conducted based on RSCU values.


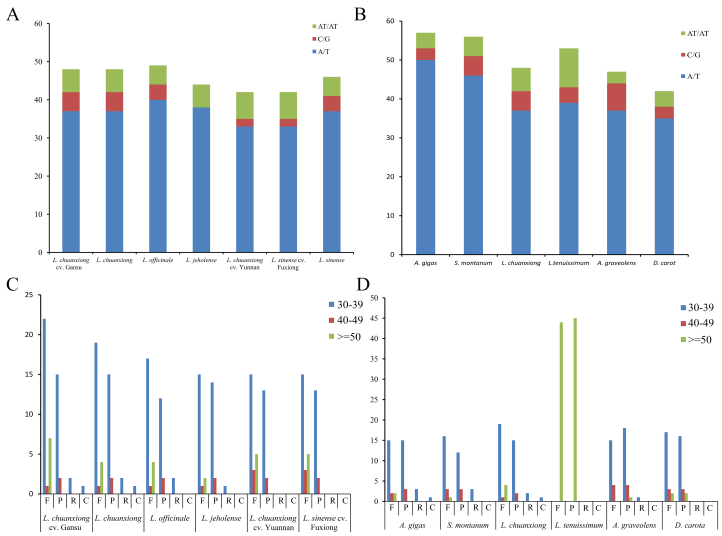


**Figure S2.** Distribution and comparison of SSRs and long repeat sequences among seven CP genomes and Apioideae


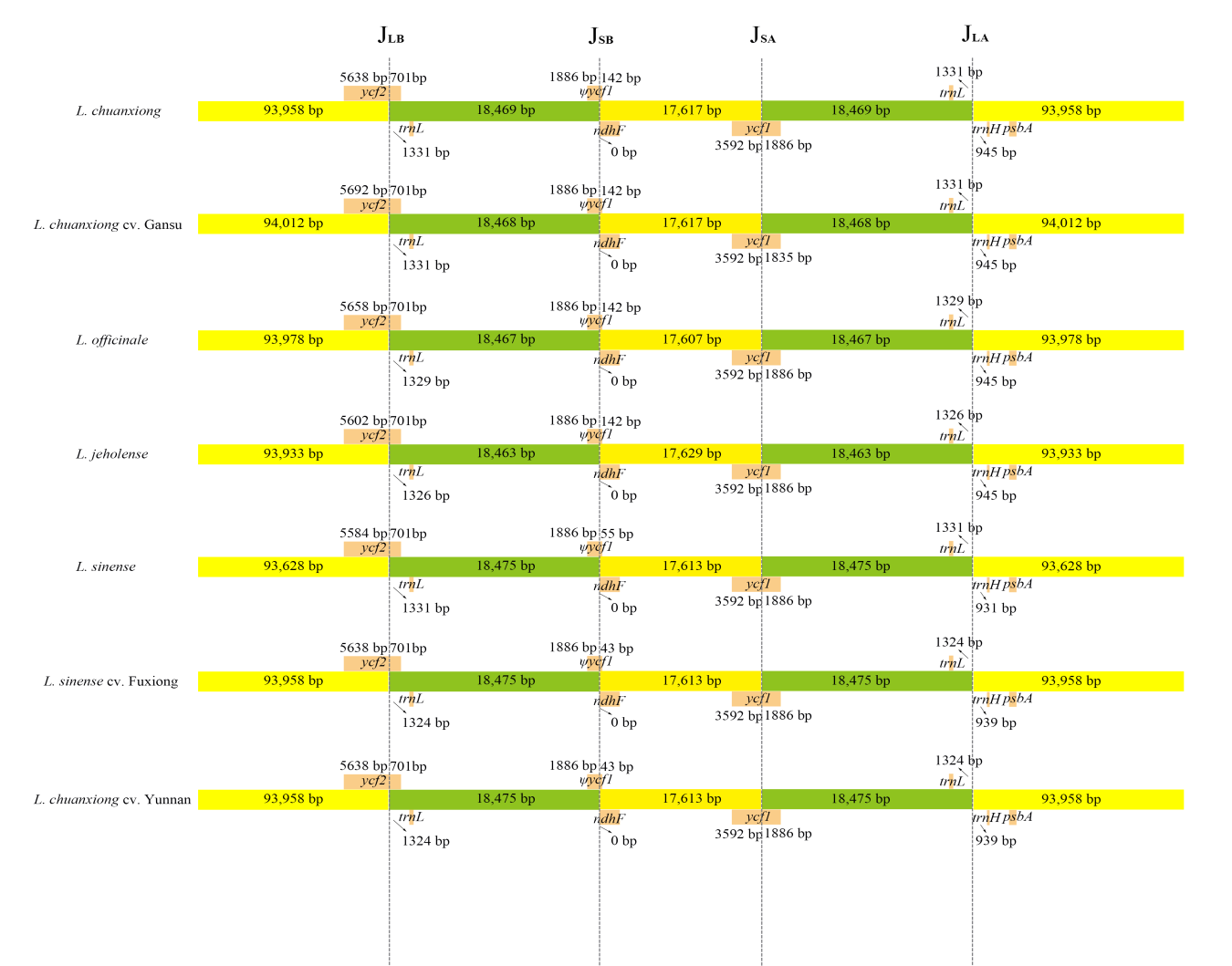


**Figure S3.** Comparison of the border positions of LSC, SSC, and IRs among seven CP genomes. J_LB_ (IRb /LSC), J_SB_ (IRb/SSC), J_SA_ (SSC/IRa), and J_LA_ (IRa/LSC) denote the junction between each corresponding region. Genes and their locations were showed using boxes with corresponding names and with ψ representing the pseudogenes. Genes transcribed clockwise and counterclockwise are presented above and below each component, respectively. The distance between the end or start coordinate of a given gene and the border sites are indicated. These features are not to scale.


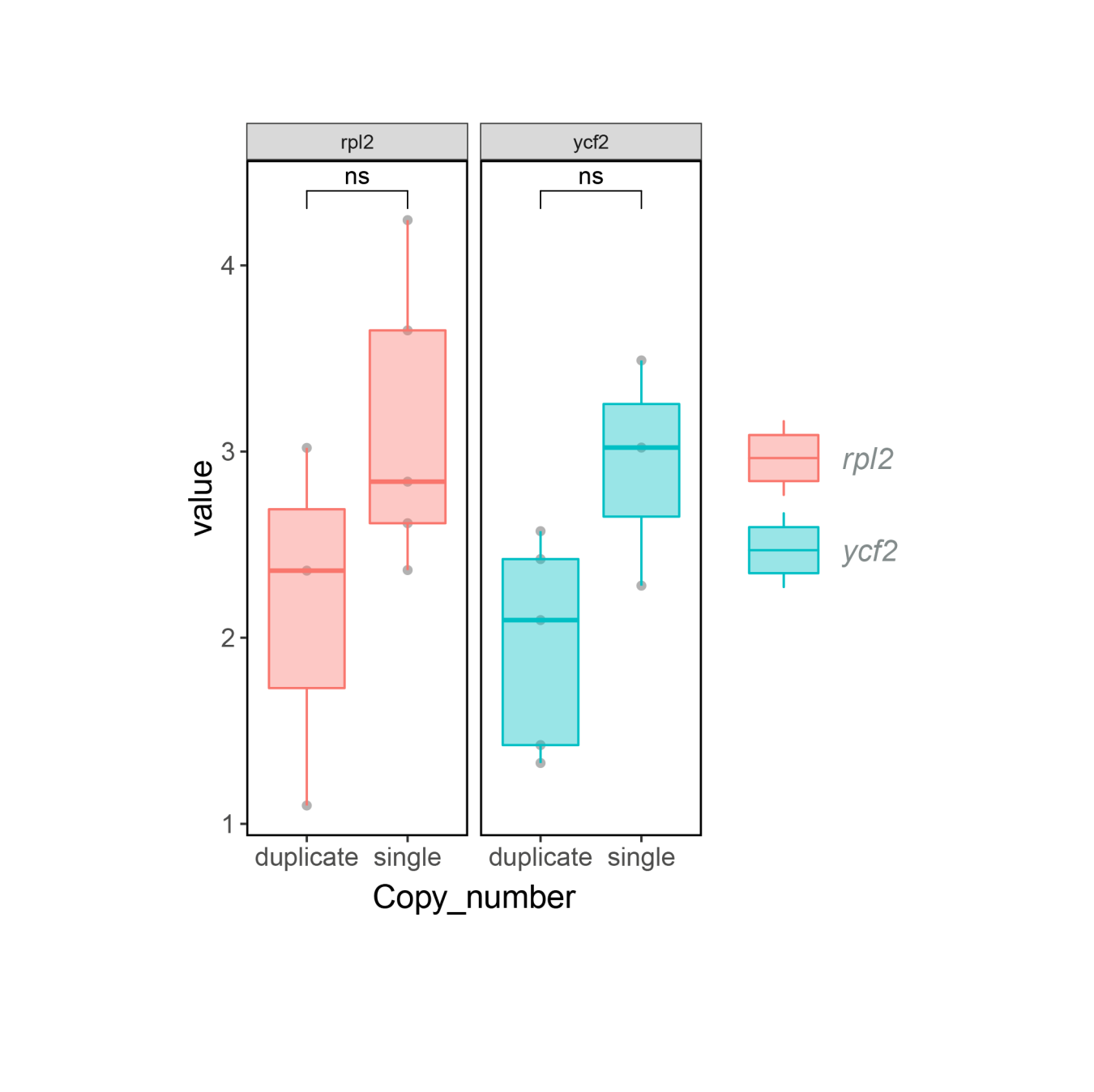


**Figure S4.** Comparison of synonymous rates of single and duplicated genes. Ns signifies no significant differences between two groups that were implemented with the Wilcoxon test. X-axis: copy numbers of genes; Y-axis: adjusted ks value.


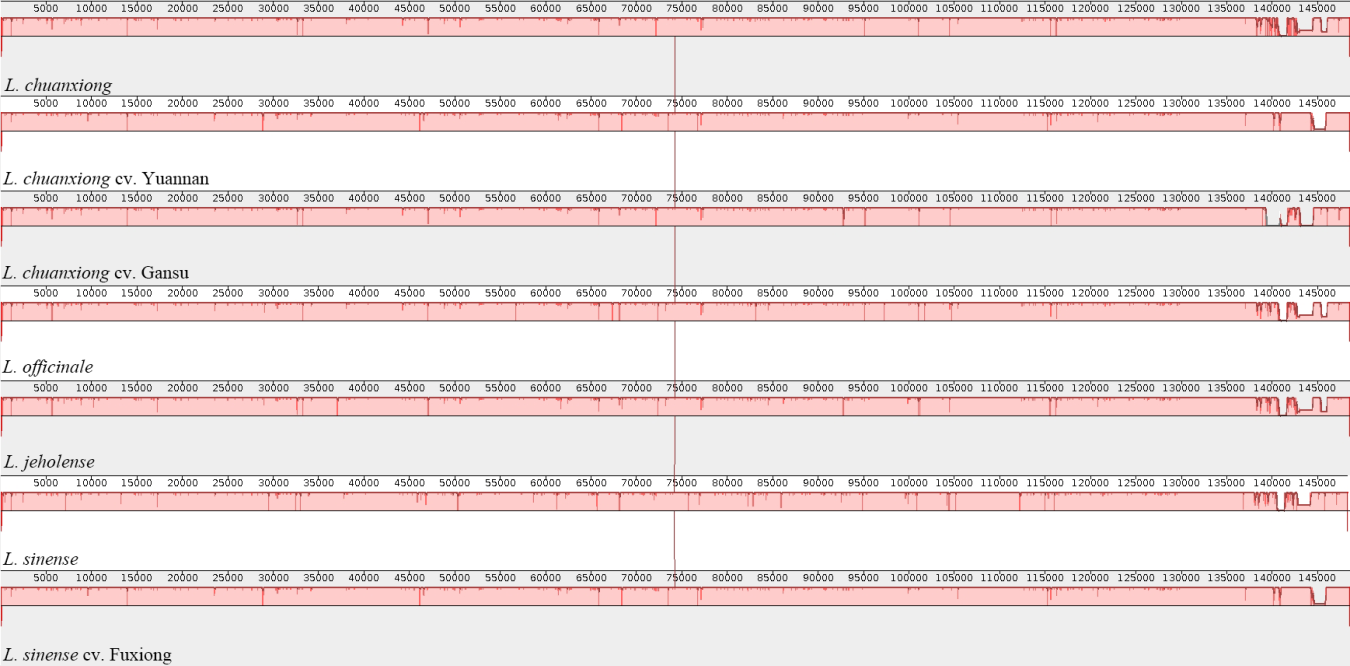


**Figure S5.** Synteny and rearrangements detected in seven CP genomes via the Mauve algorithm. A sample of 7 species are shown. Syntenic homologies were color-coded and connected by lines. Histograms within each block represent the degree of sequence similarity.


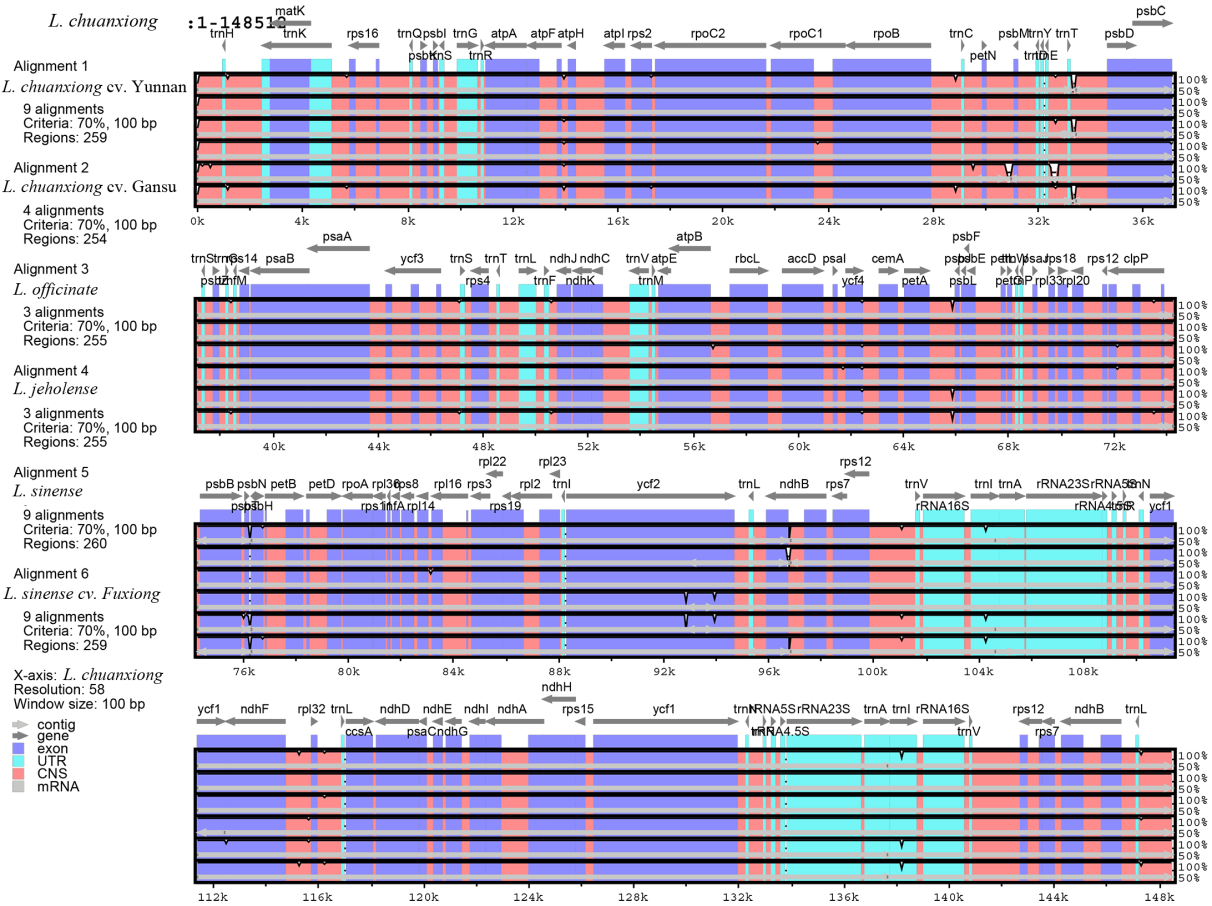


**Figure S6.** Visualization alignment of plastomes using *L. chuanxiong* as a reference genome. VISTA-based identity plots represent the sequence identity among seven CP genomes. The y-axis stands for the identity percentage ranging from 50 to 100%. Genes are denoted by grey arrows and thick black lines above the alignments, according to their orientations.


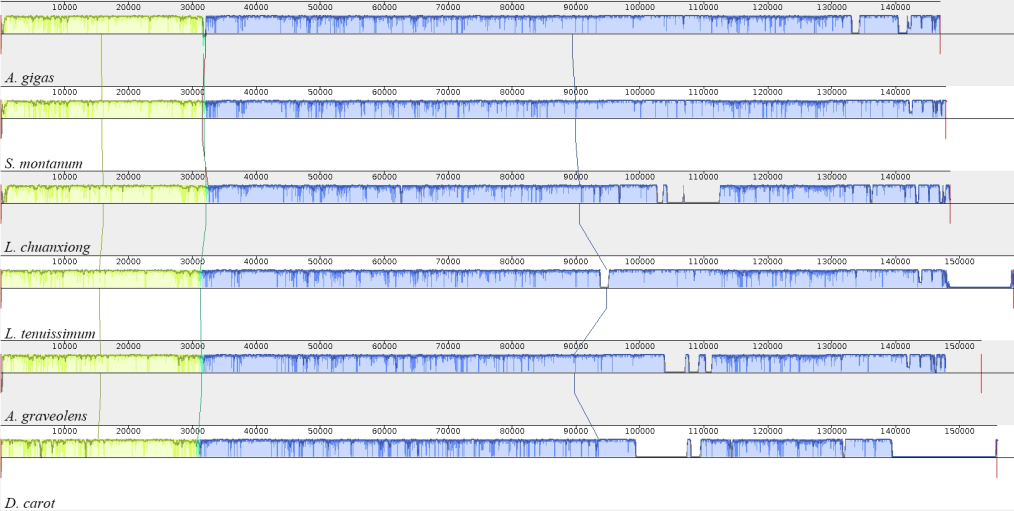


**Figure S7.** Synteny and rearrangements detected in Apiaceae CP genomes via the Mauve algorithm. A sample of 6 species are shown. Syntenic homologies were color-coded and connected by lines. Histograms within each block represent the degree of sequence similarity.


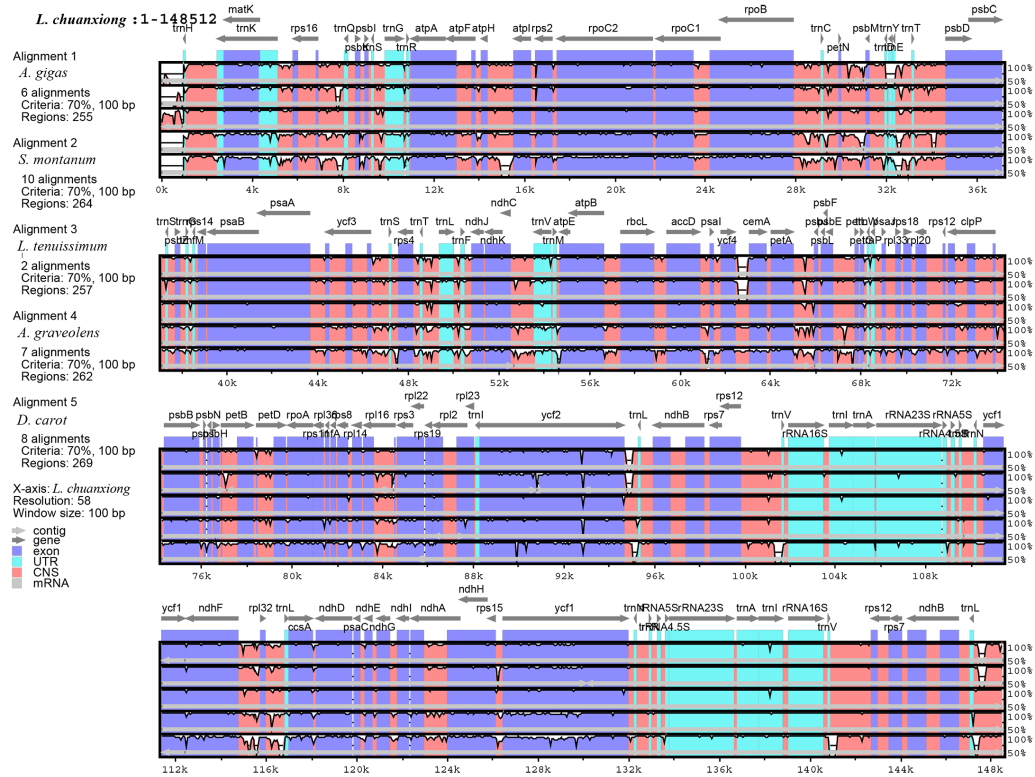


**Figure S8.** Visualization alignment of plastomes using *L. chuanxiong* as a reference genome. VISTA-based identity plots represent sequence identity among Apioideae. The y-axis stands for the identity percentage ranging from 50 to 100%. Genes are denoted by grey arrows and thick black lines above the alignments, according to their orientations.


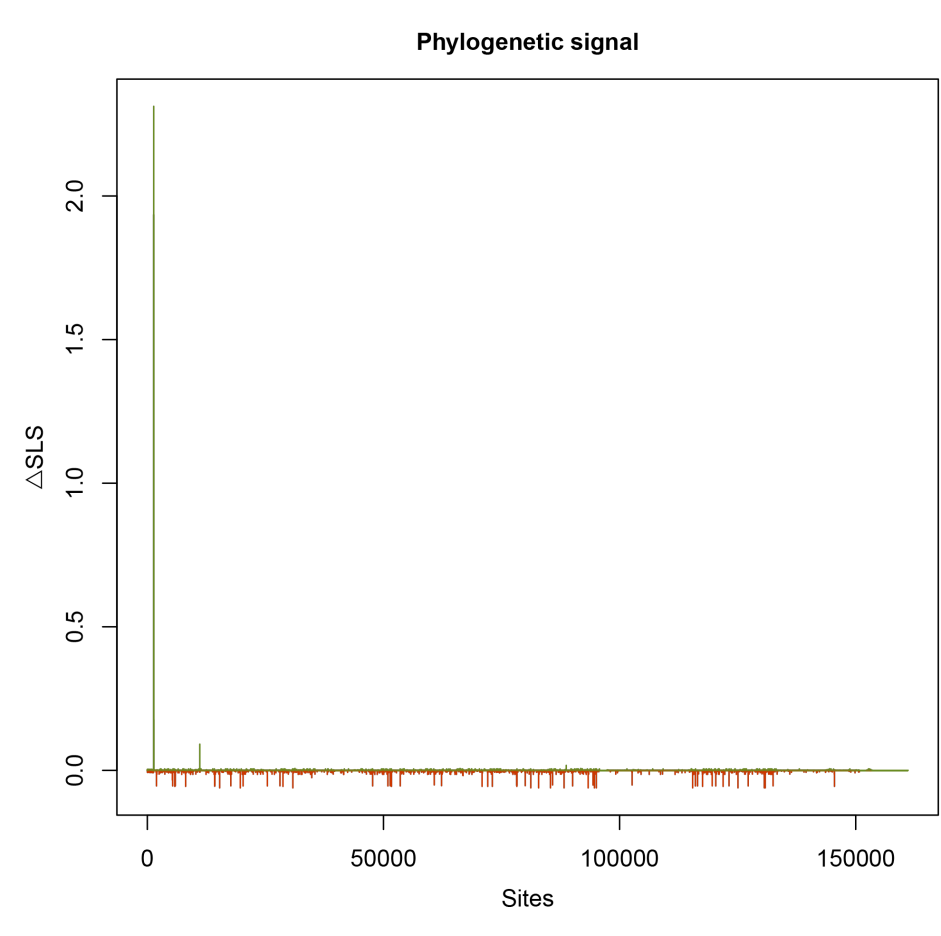


**Figure S9.** Distribution of a phylogenetic signal for the lowest support branch of the *Ligusitcum* phylogenetic tree. The X-axis is designed for sites and y-axis is designed for ΔSLS. Green bar represents the site supporting T1, whereas red bar represents the site supporting T2.
